# Hypoadiponectinemia as a marker of increased cardiovascular risk in patients with non-alcoholic fatty liver disease: correlation with albumin/creatinine ratio

**DOI:** 10.20945/2359-3997000000307

**Published:** 2020-11-09

**Authors:** Maha Assem, Mona Amin, Osama Khalafallah, Ahmed Hussien, Aasem Saif, Shrook Mousa

**Affiliations:** 1 Cairo University Internal Medicine Department Cairo Egypt Internal Medicine Department, Cairo University, Cairo, Egypt; 2 Cairo University Clinical and Chemical Pathology Department Cairo Egypt Clinical and Chemical Pathology Department, Cairo University, Cairo, Egypt

**Keywords:** Plasma adiponectin, albuminuria, carotid intima-media thickness, non-alcoholic fatty liver disease, urine albumin/creatinine ratio

## Abstract

**Objective:**

We assessed plasma adiponectin and its correlation with carotid intima-media-thickness (CIMT), as a marker of atherosclerosis, and urine albumin/creatinine ratio (ACR) in patients with non-alcoholic fatty liver disease (NAFLD).

**Subjects and methods:**

The study included 100 Egyptian subjects (50 patients with NAFLD with no history of diabetes or hypertension and 50 age and sex-matched normal healthy control subjects). Urine albumin/creatinine ratio (ACR) was assessed in all participants and fasting plasma adiponectin was measured using ELISA technique. Ultrasonography was used to diagnose NAFLD. CIMT was assessed using high-resolution Doppler ultrasonography.

**Results:**

Mild albuminuria was detected in patients with NAFLD (mean urine ACR = 42 ± 30 mg/g). Plasma adiponectin was significantly lower and urine ACR and CIMT significantly higher in patients with NAFLD as compared with the control group (P < 0.001 for all). A significant negative correlation was found between plasma adiponectin and both urine ACR and CIMT in patients with NAFLD (P < 0.001 and < 0.05 respectively). A significant positive correlation was also found between CIMT and urine ACR in those patients (P < 0.05). Plasma adiponectin and urine ACR were independent determinants of CIMT in patients with NAFLD (P < 0.01 and < 0.05 respectively).

**Conclusion:**

Patients with NAFLD, without diabetes, have an increased risk of atherosclerosis and cardiovascular disease. Hypoadiponectinemia and low-grade albuminuria are important markers of that risk.

## INTRODUCTION

Non-alcoholic fatty liver disease (NAFLD) is a chronic liver disease characterized by fat accumulation without history of viral hepatitis or heavy alcohol intake (1). Albuminuria is a well-known risk factor of both cardiovascular and chronic kidney disease (2). The relationship between NAFLD and albuminuria has been reported in many studies, but the results were inconsistent (3). Adiponectin is an adipokine abundantly produced and secreted by adipose tissues. It is widely recognized for its antidiabetic, anti-inflammatory, antiatherogenic, and cardio-protective effects (4). Adiponectin expression and its plasma levels are decreased in obese patients (5,6). It has also been reported that plasma levels of adiponectin are decreased in patients with NAFLD (7). Negative correlation between plasma adiponectin level and carotid intima-media thickness (CIMT), as a marker of atherosclerosis, was also reported in patients with type 2 diabetes (8).

In our study, we assessed plasma adiponectin and its correlation with CIMT, as a marker of atherosclerosis, and urine albumin/creatinine ratio (ACR) in patients with NAFLD.

## SUBJECTS AND METHODS

The study included 100 Egyptian subjects (55 females) aged 44-63 years (50 patients with NAFLD with no history of diabetes or hypertension and 50 age and sex-matched normal healthy control subjects). They were recruited from the internal medicine clinic at Cairo University hospital. All patients had negative hepatitis B and C viral markers. Patients with history of alcohol intake or hepatitis, high BP > 130/85 mmHg, diabetes mellitus, atherosclerotic cardiovascular disease, impaired renal function (serum creatinine higher than normal limits for age and sex), primary hyperlipidemia as well as pregnant women and smokers were excluded from the study. Primary hyperlipidemia was defined as total cholesterol > 300 mg/dL or LDL cholesterol > 190 mg/dL or triglycerides > 300 mg/dL with history of xanthomas or family history of myocardial infarction before 55 years of age (9,10). The study was approved by Cairo University ethical committee and review board. All participants provided written informed consents.

All participants were subjected to complete physical examination including blood pressure (BP) and body mass index (BMI) assessment. Laboratory investigations included serum fasting and post prandial blood glucose (FBG and PPBG), glycated hemoglobin (HbA1c), lipid profile, liver and kidney function tests and urine ACR. Fasting plasma adiponectin level was measured using a commercially available ELISA kit.

Liver ultrasound was done by conventional Bmode with a convex 3.5 MHz probe (ALT HDI ultramark machine). All participants were examined by the same operator and the same ultrasound device. NAFLD was defined as the presence of four ultrasonographic criteria: hepatorenal echo contrast, liver brightness, deep attenuation, and vascular blurring in the absence of seropositivity for hepatitis B surface antigen or antibody to hepatitis C virus, alcohol consumption, history of other causes of liver disease or medications known to produce fatty liver disease during the last six months prior to the study (11).

Measurement of CIMT was performed using high resolution color-coded Doppler ultrasonography (ALT HDI, Ultramark) using a 12 MHz linear array. All the study subjects were examined in the supine position, with the head turned 45° from the side during scanning. The reference point for the measurement of CIMT was the beginning of the dilatation of the carotid bulb, with loss of the parallel configuration of the near and far walls of the common carotid artery. The sonographer located the leading edges corresponding to the transition zones between lumen-intima and media-adventitia over a length of one cm proximal to the reference point at its thickest point, not including plaques. Plaque was identified as a localized thickened lesion (≥ 1.1 mm). All the results were the mean of the two sides. The mean CIMT of four measurements determined by B-mode ultrasound using a linear transducer (7.5-10 MHz) was calculated in each patient. Doppler examination for all participants was performed by the same skilled sonographer.

Data was transferred to the Statistical Package of Social Science Software program, version 23 (IBM SPSS Statistics for Windows, Version 23.0. Armonk, NY: IBM Corp.) to be statistically analyzed. Data were presented as mean ± standard deviation (SD. Spearman correlation coefficients (r values) were calculated to signify the association between different quantitative variables. Multivariate regression analysis was used to evaluate the independent association of risk factors. P < 0.05 was considered statistically significant.

## RESULTS

Urine ACR was significantly higher in patients with NAFLD as compared with the control group (42 ± 30 *vs* 21 ± 8 mg/g, P < 0.001). Plasma adiponectin was significantly lower in patients with NAFLD as compared with the control group (37.6 ± 20.5 *vs* 48 ± 10.5 µg/dL, P < 0.001). CIMT was significantly higher in patients with NAFLD as compared with the control group (0.10 ± 0.02 *vs* 0.08 ± 0.02 mg/g, P < 0.001). A significant negative correlation was found between plasma adiponectin and both urine ACR and CIMT (P < 0.001 and < 0.05 respectively) in patients with NAFLD. A significant positive correlation was found between CIMT and urine ACR (P < 0.05) in those patients. No significant correlation could be established between CIMT and either systolic or diastolic BPs. No significant difference was found between males and females in any of the studied parameters. Multivariate regression analysis revealed that the determinants of CIMT in patients with NAFLD were age, systolic BP, urine ACR (P < 0.05 for all) and plasma adiponectin (P < 0.01). Results of the study are summarized in Tables 1 and 2.

**Table 1:** Clinical and laboratory data of the NAFLD patients and control group

Variable	NAFLD (n = 50) Mean ± SD	Control (n = 50) Mean ± SD	P value
Age (years)	53 ± 6.1	54 ± 6.8	NS
SBP (mmHg)	125 ± 4.6	125 ± 4.5	NS
DBP (mmHg)	78 ± 4.7	77 ± 5.4	NS
BMI (kg/m^2^)	37.1 ± 6.6	35.4 ± 4.4	NS
FBS (mg/dL)	84 ± 10	83 ± 5	NS
PPBG (mg/dL)	131 ± 19	128 ± 10	NS
HbA1c (%)	5.6 ± 0.5	5.4 ± 0.7	NS
ALT (IU/L)	36 ± 19	26 ± 6	<0.01
AST (IU/L)	31 ± 18	22 ± 6	<0.01
Creatinine (mg/dL)	0.78 ± 0.16	0.76 ± 0.14	NS
Urine ACR (mg/g)	42 ± 30	21 ± 8	<0.001
Cholesterol (mg/dL)	188 ± 33	165 ± 17	<0.01
TG (mg/dL)	149 ± 76	110 ± 18	<0.001
LDL (mg/dL)	111 ± 35	99 ± 12	<0.001
HDL (mg/dL)	48 ± 10	47 ± 8	NS
Adiponectin (µg/dL)	37.6 ± 20.5	48 ± 10.5	<0.001
CIMT (mm)	0.10 ± 0.02	0.08 ± 0.02	<0.001

NAFLD: non-alcoholic fatty liver disease; T2DM: type 2 diabetes mellitus; NA: not applicable; SBP:, systolic blood pressure; DSP: diastolic blood pressure; BMI: body mass index; FBG: fasting blood glucose; PPBG: postprandial blood glucose; HbA1c: glycated hemoglobin; ALT: alanine transferase; AST: aspartate transferase; UAC: urinary albumin creatinine ratio; TG: triglycerides; LDL: low-density lipoprotein; HDL: high density lipoprotein; CIMT: carotid intima-media thickness; SD: standard deviation; NS: non-significant. P < 0.05 is statistically significant.

**Table 2:** Determinants of CIMT in patients with NAFLD (multivariate regression analysis)

Variable	P value
Age	<0.05
SBP	<0.05
DBP	NS
BMI	NS
FBS	NS
PPBG	NS
HbA1c	NS
ALT	NS
AST	NS
Creatinine	NS
Urine ACR	<0.05
Cholesterol	NS
TG	NS
LDL	NS
HDL	NS
Adiponectin	< 0.01

CIMT: carotid intima-media thickness; NAFLD: non-alcoholic fatty liver disease; SBP: systolic blood pressure; DSP: diastolic blood pressure; BMI: body mass index; FBG: fasting blood glucose; PPBG: postprandial blood glucose; HbA1c: glycated hemoglobin; ALT: alanine transferase; AST: aspartate transferase; UAC: urinary albumin creatinine ratio; TG: triglycerides; LDL: low-density lipoprotein; HDL: high density lipoprotein; NS: non-significant. P < 0.05 is statistically significant.

## DISCUSSION

NAFLD is associated with dysregulation of metabolic and inflammatory pathways which can lead to extrahepatic manifestations involving the kidney (12). The results of the previous research work on the relationship between NAFLD and albuminuria have been inconsistent (3). Adiponectin plays a pivotal role in energy metabolism. Its plasma level is decreased in obesity and increases after weight loss (5,13).

We assessed plasma adiponectin and urine ACR and their correlation with CIMT, as a marker of atherosclerosis, in patients with NAFLD without diabetes mellitus. Our results revealed low level albuminuria in patients with NAFLD. Urine ACR was significantly higher in patients with NAFLD as compared with the control group. Kang and cols. also reported that patients with NAFLD, without diabetes, had low-grade albuminuria, but they included only men in their study (12). Lin and cols. also found that NAFLD was significantly associated with an increased risk of low-grade albuminuria in middle-aged and elderly Chinese men (14).

Our study showed that plasma adiponectin was significantly lower in patients with NAFLD as compared with control subjects. These results agree with those of Bugianesi and cols. who reported significantly lower plasma adiponectin levels in patients with NAFLD as compared with healthy control subjects in Italy, but their study participants were mainly males (7). Fadaei and cols. also reported a lower adiponectin level in Iranian patients with NAFLD as compared with a control group (15).

A significant negative correlation was found between plasma adiponectin and urine ACR in our study group. Atta and cols. found an association between microalbuminuria and adiponectin in obese non-diabetic individuals (16). Ahima also reported that Obese African Americans had reduced adiponectin levels associated with albuminuria (17). Kacso and cols. reported that lower plasma adiponectin levels seem to be predictive of increased urine ACR in patients with type 2 diabetes (18). Serum adiponectin was found to inversely correlate with albuminuria in men with essential hypertension (19). The association between plasma adiponectin and kidney disease is rather complex. The inverse correlation between adiponectin and low-grade albuminuria may suggest that adiponectin deficiency has a causative role in abnormal glomerular function (20,21).

CIMT has been reported to be a representative of subclinical and asymptomatic atherosclerotic vascular diseases. It correlates with the extent of atherosclerotic lesions elsewhere in the body (22-24). In our study, CIMT was significantly higher in patients with NAFLD as compared with the control group. Our results agree with those of Fracanzani and cols. who reported a significant increase in CIMT in patients with NAFLD as compared with normal control subjects (25).

No significant correlation could be established, in our study between, CIMT and either systolic or diastolic BPs. But multivariate regression analysis showed that age and systolic BP were independent determinants of CIMT in patients NAFLD. Alizargar and Bai reported that age, waist circumference, systolic BP and HbA1C were determinants of CIMT in 331 subjects from a community-based prospective cohort study (26).

Our study also revealed a significant positive correlation between CIMT and urine ACR in patients with NAFLD. Urine ACR was a strong determinant of CIMT in our study group. In an Iranian study, Shahrokh and cols. found a significant relationship between CIMT and renal parameters including albuminuria and estimated glomerular filtration rate in patients with type 2 diabetes (27). Similar results were reported in India by Gayathri and cols. (28).

Our results showed a significant negative correlation between plasma adiponectin and CIMT in the study group. Our study also revealed that plasma adiponectin was a strong determinant of CIMT in patients with NAFLD. Fadaei and cols. reported a negative a correlation between plasma adiponectin and CIMT in 49 Iranian patients with NAFLD (15). de Almeida-Pititto and cols. reported a significant inverse correlation between plasma adiponectin and CIMT in non-diabetic individuals with no cardiovascular disease in Brazil. (29). Shargorodsky and cols. reported an inverse relation between plasma adiponectin and CIMT in 47 obese non-diabetic Israeli individuals (30). However, the last two studies did not include patients with NAFLD.

Despite the important results of the study, we are aware of its limitations. The rather small number of the study group and the effect of dyslipidemia on CIMT are major ones. A larger study to assess the correlation of plasma adiponectin and urine ACR with CIMT in dyslipidemic and non-dyslipidemic patients with NAFLD will help to explore the antiatherogenic role of adiponectin and the value of albuminuria as a risk indicator in those patients.

In conclusion, low level albuminuria is detected in patients with NAFLD without diabetes. There is a negative correlation between plasma adiponectin and both urine ACR and CIMT in patients with NAFLD. There is also a significant positive correlation between CIMT and urine ACR in those patients. Urine ACR and Plasma adiponectin are independent determinants of CIMT in patients with NAFLD. Patients with NAFLD, without diabetes, have an increased risk of atherosclerosis. Hypoadiponectinemia and low-grade albuminuria are important indicators of that risk.

## References

[1] Nascimbeni F, Pais R, Bellentani S, Day CP, Ratziu V, Loria P (2013). From NAFLD in clinical practice to answers from guidelines. J Hepatol.

[2] Kidney Disease. Improving Global Outcomes (KDIGO) Blood Pressure Work Group (2012). KDIGO Clinical Practice Guideline for the Management of Blood Pressure in Chronic Kidney Disease. Kidney Int Suppl.

[3] Wijarnpreecha K, Thongprayoon C, Boonpheng B, Panjawatanan P, Sharma K, Ungprasert P (2018). Nonalcoholic fatty liver disease and albuminuria: a systematic review and meta-analysis. Eur J Gastroenterol Hepatol.

[4] Ohashi K, Ouchi N, Matsuzawa Y (2012). Anti-inflammatory and anti-atherogenic properties of adiponectin. Biochimie.

[5] Daniele A, Cammarata R, Masullo M, Nerone G, Finamore F, D’Andrea M (2008). Analysis of adiponectin gene and comparison of its expression in two different pig breeds. Obesity (Silver Spring).

[6] De Rosa A, Monaco ML, Capasso M, Forestieri P, Pilone V, Nardelli C (2013). Adiponectin oligomers as potential Indicators of adipose tissue improvement in obese subjects. Eur J Endocrinol.

[7] Bugianesi E, Pagotto U, Manini R, Vanni E, Gastaldelli A, de Iasio R (2005). Plasma adiponectin in nonalcoholic fatty liver is related to hepatic insulin resistance and hepatic fat content, not to liver disease severity. J Clin Endocrinol Metab.

[8] Dullaart RP, de Vries R, van Tol A, Sluiter WJ (2007). Lower plasma adiponectin is a marker of increased intima-media thickness associated with type 2 diabetes mellitus and with male gender. Eur J Endocrinol.

[9] Austin MA, Hutter CM, Zimmern RL, Humphries SE (2004). Genetic causes of monogenic heterozygous familial hypercholesterolemia: a HuGE prevalence review. Am J Epidemiol.

[10] Yuan G, Al-Shali KZ, Hegele RA (2007). Hypertriglyceridemia: its etiology, effects and treatment. CMAJ.

[11] Eshraghian A, Dabbaghmanesh MH, Eshraghian H, Fattahi MR, Omrani GR (2013). Nonalcoholic fatty liver disease in a cluster of Iranian population: thyroid status and metabolic risk factors. Arch Iran Med.

[12] Kang SH, Cho KH, Do JY (2019). Non-alcoholic fatty liver disease is associated with low-grade albuminuria in men without diabetes mellitus. Int J Med Sci.

[13] Meyer LK, Ciaraldi TP, Henry RR, Wittgrove AC, Phillips SA (2013). Adipose tissue depot and cell size dependency of adiponectin synthesis and secretion in human obesity. Adipocyte.

[14] Lin L, Lu J, Huang X, Ding L, Huang Y, Wang P (2016). Nonalcoholic fatty liver disease is associated with low-grade albuminuria in Chinese adults. QJM.

[15] Fadaei R, Meshkani R, Poustchi H, Fallah S, Moradi N, Panahi G (2019). Association of carotid intima media thickness with atherogenic index of plasma, apo B/apo A-I ratio and paraoxonase activity in patients with non-alcoholic fatty liver disease. Arch Physiol Biochem.

[16] Atta MI, Abdalla NH, Ibrahim AA (2016). Microalbuminuria and adiponectin in obese non-diabetic non-hypertensive people. Egypt J Obes Diabetes Endocrinol.

[17] Ahima RS (2008). Linking adiponectin to proteinuria. J Clin Invest.

[18] Kacso I, Lenghel A, Bondor CI, Moldovan D, Rusu C, Nita C (2012). Low plasma adiponectin levels predict increased urinary albumin/creatinine ratio in type 2 diabetes patients. Int Urol Nephrol.

[19] Tsioufis C, Dimitriadis K, Chatzis D, Vasiliadou C, Tousoulis D, Papademetriou V (2005). Relation of microalbuminuria to adiponectin and augmented C-reactive protein levels in men with essential hypertension. Am J Cardiol.

[20] Sharma K, Ramachandrarao S, Qiu G, Usui HK, Zhu Y, Dunn SR (2008). Adiponectin regulates albuminuria and podocyte function in mice. J Clin Invest.

[21] Ix JH, Sharma K (2010). Mechanisms linking obesity, chronic kidney disease, and fatty liver disease: the roles of fetuin-A, adiponectin, and AMPK. J Am Soc Nephrol.

[22] Stein JH, Korcarz CE, Hurst RT, Lonn E, Kendall CB, Mohler ER (2008). American Society of Echocardiography Carotid Intima-Media Thickness Task Force. Use of carotid ultrasound to identify subclinical vascular disease and evaluate cardiovascular disease risk: A consensus statement from the American Society of Echocardiography Carotid Intima-Media Thickness Task Force. Endorsed by the Society for Vascular Medicine. J Am Soc Echocardiogr.

[23] Liviakis L, Pogue B, Paramsothy P, Bourne A, Gill EA (2010). Carotid intima-media thickness for the practicing lipidologist. J Clin Lipidol.

[24] Society of Atherosclerosis Imaging and Prevention Developed in collaboration with the International Atherosclerosis Society (2011). Appropriate use criteria for carotid intima media thickness testing. Atherosclerosis.

[25] Fracanzani AL, Burdick L, Raselli S, Pedotti P, Grigore L, Santorelli G (2008). Carotid artery intima-media thickness in nonalcoholic fatty liver disease. Am J Med.

[26] Alizargar J, Bai CH (2018). Factors associated with carotid Intima media thickness and carotid plaque score in community-dwelling and non-diabetic individuals. BMC Cardiovasc Disord.

[27] Shahrokh H, Zakerkish M, Jenabi A, Hanafi MG, Rahim F, Rezazadeh A (2015). Association of Microalbuminuria and Estimated Glomerular Filtration Rate with Carotid Intima-Media Thickness in Patients With Type 2 Diabetes Mellitus. Jundishapur J Health Res.

[28] Gayathri R, Chandni R, Udayabhaskaran V (2012). Carotid artery intima media thickness in relation with atherosclerotic risk factors in patients with type 2 diabetes mellitus. J Assoc Physicians India.

[29] de Almeida-Pititto B, Ribeiro FF, Santos IS, Lotufo PA, Bensenor IM, Ferreira SR (2017). Association between carotid intima-media thickness and adiponectin in participants without diabetes or cardiovascular disease of the Brazilian Longitudinal Study of Adult Health (ELSA-Brasil). Eur J Prev Cardiol.

[30] Shargorodsky M, Boaz M, Goldberg Y, Matas Z, Gavish D, Fux A (2009). Adiponectin and vascular properties in obese patients: is it a novel biomarker of early atherosclerosis?. Int J Obes (Lond).

